# Early and specific detection of Alzheimer’s disease: more than a (virtual) reality?

**DOI:** 10.1093/braincomms/fcae014

**Published:** 2024-02-12

**Authors:** Osborne F X Almeida

**Affiliations:** Max Planck Institute of Psychiatry, 80804 Munich, Germany; School of Medicine, University of Minho, 4710-057 Braga, Portugal

## Abstract

This scientific commentary refers to ‘Path integration deficits are associated with phosphorylated tau accumulation in the entorhinal cortex’, by Koike *et al*. (https://doi.org/10.1093/braincomms/fcad359).

**This scientific commentary refers to ‘Path integration deficits are associated with phosphorylated tau accumulation in the entorhinal cortex’, by Koike *et al*. (https://doi.org/10.1093/braincomms/fcad359).**

Spatial navigation—determining and maintaining a trajectory from one place to another by recognition of spatial position by accumulating self-motion cues—is essential for independent daily living. Spatial navigation requires continuous switching between body-centred (egocentric) and object-to-object (allocentric) strategies.^1^ Whereas the basal ganglia and parietal lobe support egocentric spatial representations, allocentric representations depend on the hippocampus and entorhinal cortex in the medial temporal lobe. Grid cells in the entorhinal cortex play a crucial role in path integration by facilitating the use of self-motion cues to estimate distance travelled and angular displacement and, therefore, position in the environment. These cells are innervated by the visual cortex and send projections to place cells in the CA1 subfield of the hippocampus. In the context of Alzheimer’s disease, it is important to note that the entorhinal cortex is the first brain area to develop tau-containing neurofibrillary tangle (NFT) lesions.^2^

Impaired judgement of distances and formation of mental maps results in difficulties in spatial orientation and navigation. Spatial disorientation is often experienced by patients with pre-symptomatic Alzheimer’s disease and mild cognitive impairment,^3^ but navigational skills may also be impaired in otherwise cognitively intact elderly subjects who find themselves in a non-familiar environment^4^; this is supposedly due to greater bias towards egocentric versus allocentric strategies and translating between them. Despite evidence that impediments in spatial navigation are specific to Alzheimer’s disease,^5^ most clinical assessments are still largely based on episodic memory, a measure that is not specific to Alzheimer’s disease.^6^

The protracted and insidious nature of Alzheimer’s disease pathology, and unlikelihood of its reversibility, makes it futile to presently contemplate ‘cures’ for the disease. Rather, timely pharmacological and non-pharmacological intervention is a more realistic goal; but this goal can only be achieved if pathology can be predicted or detected sufficiently early. To this end, the last two decades has seen the development of stand-alone or combined objective markers of incipient Alzheimer’s disease based on neuropsychological assessment, neuroimaging, genomics and fluid biomarker screening. However, consensus on the ‘perfect’ individual or set of early predictors is still lacking.

Recent studies have begun to explore the potential of digital biomarkers for early diagnosis of Alzheimer’s disease through the use of head-mounted immersive virtual reality (iVR), devices^7-9^ (defined as ‘systems that encompass the user’s visual field and where virtual movement replicates actual head or bodily movement’^10^). One of these showed that iVR can discriminate between healthy aged subjects and patients with mild cognitive impairment^8^ and observed that ‘path integration impairment in early Alzheimer’s disease is not an extension of healthy aging’ (cf. Li and King^4^).

The work by Koike *et al*.^7^ in this issue of *Brain Communications* supports the notion that detection of deficits in path integration may help identify persons likely to develop Alzheimer’s disease. Given that spatial navigation abilities begin to decline during asymptomatic and prodromal stages of Alzheimer’s disease, the authors suggest that cohort selection and post-treatment assessment based on objective and quantifiable criteria in future clinical trials are more likely to generate clearer results than those obtained to date.

Importantly, Koike *et al.*^7^ show that their iVR paradigm distinguishes between entorhinal cortex-dependent path integration and hippocampus-dependent spatial memory and that errors in path integration increase with age; by extrapolation from the Braak age-related classification, significant declines in path integration ability would start from around 50 years of age. This observation is consistent with the authors’ assertion that preventative or therapeutic interventions for Alzheimer’s disease should be initiated at Braak stages I and II when NFT are confined to the entorhinal cortex^2^ if they are to be efficacious.

While the authors^7^ did not directly measure NFT in their subjects, they ingeniously ‘borrowed’ from Braak’s neuropathological staging of Alzheimer’s disease as a function of age and appearance of NFT in the entorhinal cortex.^2^ Supporting their deductions, they showed that chemogenetic inactivation of entorhinal cortex neurons disrupted path integration in healthy young adult mice. In addition, using a modified L-maze, they showed that path integration is impaired in middle-aged tau-overexpressing transgenic mice, coinciding with the appearance of NFT in the entorhinal cortex. From comparison of the performance of mice in the Morris water maze versus the L-maze, the authors confirmed that NFT accumulation in the entorhinal cortex interferes with path integration but not spatial memory. The latter is consistent with the conclusions of one of the other iVR-based studies,^8^ namely, that deficits in path integration appear in at-risk individuals before conversion to pre-clinical Alzheimer’s disease. An interesting question to be addressed in future research concerns the nature (e.g. connectivity) of the influence of NFT in the entorhinal cortex (allocentric behaviour) on basal ganglia (egocentric behaviour) since these brain regions inter-dependently contribute to the efficacy of path integration.

Despite the limitations of this initial research by Koike *et al.*^7^ (also see Newton *et al*.^8^ and Castegnaro *et al*.^9^), the work deserves credit for bringing iVR-based assessment of spatial navigation for early detection of Alzheimer’s disease closer to reality. However, much work is still needed before their findings can be translated into a robust diagnostic tool. For the purpose of diagnosis, the findings will require replication and validation in larger cohorts. Since a multiplicity of factors place individuals at risk for developing Alzheimer’s disease, subjects must be screened and selected on the basis of objective neuropsychological, neuroimaging, genetic and fluid biomarker criteria, with less reliance on self-reports of physical, mental and cognitive health status. Further, besides age, data should be stratified to include a broader set of demographic characteristics—for example, the prevalence of Alzheimer’s disease is higher in women than in men (and there are sex differences in spatial and other cognitive functions), and higher socioeconomic and educational status are reported to have a mitigating effect on the time of disease onset. Lastly, to avoid biases in cohort selection, investigators need to consider that volunteers’ motivation to enrol in clinical studies may be confounded by factors such as family history of dementia, education level and general stress, mood and anxiety levels.

There is broad consensus that proof of the success of new preventative and ameliorating therapies for Alzheimer’s disease depends on early detection of the disease by robust analytical tools that are sensitive, accurate, standardizable, generalizable and affordable. At least until sufficient data become available, diagnostic approaches should ideally include analysis of areas of the brain in which neuropathology originates and spreads from. While PET represents the gold standard for monitoring late-stage Alzheimer’s disease when tau proteinopathy is present (the so-called T_2_ category of biomarkers; Braak stages III and VI), there is presently no suitable approach for monitoring pre-tangle tau (T_1_) *in situ*. The development of better early diagnostics clearly demands even greater multi-disciplinarity effort.

The introduction of iVR to the test battery for early diagnosis of Alzheimer’s disease has the potential to greatly advance the search for effective therapies. As reviewed in detail,^10^ iVR gives both insight into cognitive function in a quantifiable manner and information about the processing and integration of sensory cues. Several features of iVR make its use widely applicable whether in clinics, practices or homes: the instrumentation is cheap and easy to use, and the software can be standardized to enable longitudinal follow-up examinations and comparison of data across centres. Importantly, iVR yields ecologically valid data, allowing testing in large landscapes without demands on space. From the patient perspective, iVR technology is intuitive and well-tolerated by elderly persons. Many older people reportedly find iVR to be a rewarding experience, although a minority are susceptible to cybersickness or may be anxious about new technologies.

In conclusion, the recent work by Koike *et al.*^7^ and others^8,9^ opens a promising new phase in the diagnosis and management of Alzheimer’s disease—from sufficiently early detection to the development of properly targeted treatments.

## References

[1] Lithfous S, Dufour A, Després O. Spatial navigation in normal aging and the prodromal stage of Alzheimer's disease: Insights from imaging and behavioral studies. Ageing Res Rev. 2013;12(1):201–213. 22771718 10.1016/j.arr.2012.04.007

[2] Braak H, Braak E. Staging of Alzheimer's disease-related neurofibrillary changes. Neurobiol Aging. 1995;16(3):271–278. discussion 278-284. 7566337 10.1016/0197-4580(95)00021-6

[3] Serino S, Cipresso P, Morganti F, Riva G. The role of egocentric and allocentric abilities in Alzheimer's disease: A systematic review. Ageing Res Rev. 2014;16:32–44. 24943907 10.1016/j.arr.2014.04.004

[4] Li AWY, King J. Spatial memory and navigation in ageing: A systematic review of MRI and fMRI studies in healthy participants. Neurosci Biobehav Rev. 2019;103:33–49. 31129234 10.1016/j.neubiorev.2019.05.005

[5] Tu S, Wong S, Hodges JR, Irish M, Piguet O, Hornberger M. Lost in spatial translation—A novel tool to objectively assess spatial disorientation in Alzheimer's disease and frontotemporal dementia. Cortex. 2015;67:83–94. 25913063 10.1016/j.cortex.2015.03.016

[6] Coughlan G, Laczó J, Hort J, Minihane AM, Hornberger M. Spatial navigation deficits—Overlooked cognitive marker for preclinical Alzheimer disease? Nat Rev Neurol. 2018;14:496–506. 29980763 10.1038/s41582-018-0031-x

[7] Koike R, Soeda Y, Kasai A, et al Path integration deficits are associated with phosphorylated tau accumulation in the entorhinal cortex. Brain Comm. 2024:fcad359. 10.1093/braincomms/fcad359 PMC10859636 38347945

[8] Newton C, Pope M, Rua C, et al Path integration selectively predicts midlife risk of Alzheimer's disease. *bioRxiv*. 2023. 10.1101/2023.01.31.526473 PMC11032581 38421123

[9] Castegnaro A, Ji Z, Rudzka K, Chan D, Burgess N. Overestimation in angular path integration precedes Alzheimer's dementia. Curr Biol. 2023;33(21):4650–4661. e7.37827151 10.1016/j.cub.2023.09.047

[10] Clay F, Howett D, FitzGerald J, Fletcher P, Chan D, Price A. Use of immersive virtual reality in the assessment and treatment of Alzheimer's disease: A systematic review. J Alzheimers Dis. 2020;75(1):23–43. 32280091 10.3233/JAD-191218 PMC7306888

